# Investigation of Structural, Dielectric and Optical Properties of Polyaniline—Magnesium Ferrite Composites

**DOI:** 10.3390/nano13152234

**Published:** 2023-08-02

**Authors:** Priyanka Kolhar, Basavaraja Sannakki, Meenakshi Verma, Siddaramappa Suresha, Mansoor Alshehri, Nehad Ali Shah

**Affiliations:** 1 Department of Physics, Gulbarga University, Kalaburgi 585106, Karnataka, India; priyankakolhar25@gmail.com (P.K.); sannakki.phy@gmail.com (B.S.); 2 University Centre for Research and Development, Chandigarh University, Gharuan, Mohali 160055, Punjab, India; 3 Department of Physics, Government First Grade College, Holalkere 577552, Karnataka, India; sureshafgc@gmail.com; 4 Department of Mathematics, College of Sciences, King Saud University, P.O. Box 2455, Riyadh 11451, Saudi Arabia; mhalshehri@ksu.edu.sa; 5 Department of Mechanical Engineering, Sejong University, Seoul 05006, Republic of Korea

**Keywords:** Polyaniline, ferrites, nanoparticles, composites synthesis, solution combustion method, optical properties, dielectric properties

## Abstract

A study on the influence of magnesium ferrite nanoparticles on the optical and dielectric attributes of Polyaniline has been conducted. Magnesium nano Ferrite powder is synthesized by the self-propagating solution combustion method. Polyaniline–Magnesium nano ferrite composites are synthesized by chemical oxidative polymerization of aniline with the addition of Magnesium nanoparticles. The samples are characterized with XRD and UV-Vis spectrometer, in the wavelength range of 200–800 nm and studied for optical properties. Dielectric properties are studied in the frequency range of 50 Hz to 5 MHz. X-ray diffraction reveals single phase formation of Magnesium ferrite, whereas Polyaniline shows an amorphous nature. In the XRD of the composites, we see the crystalline peaks of ferrite becoming more intense with the addition of ferrite and whereas the peak of Polyaniline diminishes. The crystallite size is quantified with the Debye—Scherrer formula, and it increases as the content of ferrite in the composites increases. The micro-strain decreases in the composites as the percentage of ferrite enhances in the composites. In the UV-Vis absorption spectra of composites, the peaks of Polyaniline shift to higher wavelength and there is also an absorption band in the spectra of composites corresponding to that of Magnesium ferrite particles. Both direct and indirect band gaps are calculated with the Tauc plot, and both the optical band gap decrease as the percentage of ferrite increases in the composite. The dielectric loss and dielectric constant both decrease with frequency in all the samples, and the dielectric response are in good agreement with Maxwell—Wagner model. Ferrite—polymer composites with both conducting and magnetic properties are considered useful for electromagnetic shielding and microwave absorption.

## 1. Introduction

The research at nanoscale composites is primarily focusing on developing materials with innovative combined properties not demonstrated by individual components. These properties are dependent on the morphology and interfacial characteristics of their components. Polymers with suitable fillers serve the purpose of materials with synergistic properties. Among the polymers, Polyaniline, poly-pyrrole and polythiophene are studied as they are intrinsically conducting in nature. Polyaniline has received a lot of attention owing to its easy preparative method, high stability and very good conductivity, which is tunable depending on the changing degree of oxidation and protonation [1]. The conductivity of Polyaniline ranges from that of an insulator to a semiconductor depending on the synthesis method; as in Polyaniline, between the repeating moieties of benzenoid and quinoid parts, there is nitrogen which can be protonated and deprotonated. Polyaniline can be prepared by methods such as Chemical oxidative polymerization, electrochemical synthesis, vacuum evaporation, photopolymerization and plasma polymerization. However, in the chemical oxidation method, we obtain Polyaniline powder which can be produced in bulk. More research is happening in the growth of polymer doped with organic or inorganic material mainly to boost the desirable properties of the composites, and these composites possess flexible processability properties of a polymer combined with desired properties exhibited by fillers. The inorganic filler materials have a wide range from metal oxides, nitrates, carbonates etc. One of the efficient inorganic additives for the polymer to boost optical and magnetic properties are ferrites. In Ferrites, spinel ferrites form a group of ferrites with the general formula MFe_2_0_4_ wherein M is a divalent metal (M = Mg, Ni, Zn, Mn, etc.). The properties of spinel ferrites at the nanoscale are tailored for many applications, and these depend on the preparation method, the sintering temperature, time and other factors [2,3,4,5]. Nowadays, research is focused on the composites of Polyaniline with inorganic fillers such as ferrite and in these composites, we see both electrical conduction as well as magnetic properties, along with added advantages of improved thermal stability and mechanical strength [6]. These rare properties of Polyaniline—Ferrite composites find important applications in the emerging electronic world, such as microwave absorption [7,8] and Electromagnetic shielding [9]. Magnesium ferrite [MF] is a soft spinel ferrite. Magnesium ferrite is a very useful and widely studied ferrite with applications such as in Biomedical [10] environmental pollution removal [11,12], gas sensor [13], remediator of Congo-red [14] etc. Many optical applications require fine-tuning of the optical properties of Polyaniline nanocomposites. Magnesium ferrite has good chemical and thermal stability, making it a suitable material for use as a substrate withstanding high temperatures in optical applications [15]. Henaish AM et al. [16] studied the Optical parameters for polypyrrole—NiAl_x_Fe_2-x_O_4_ nanocomposites wherein the optical band gap decreased with the addition of Al in the composites. Indrakanti R et al. [17] studied the optical parameter of GaNFe_2_O_3_–PPY, where the band gap decreased with increasing content of gallium nieetride ferrite in the composite. Parvathi K et al. [18] analyzed the optical features of zinc ferrite (ZnFe_2_O_4_)/Chlorinated natural rubber (Cl-NR) in which the addition of zinc ferrite to chlorinated natural rubber decreased its optical energy band gap. Based on the promising literature survey of polymer—ferrite composites, Saini M et al. [19] studied the electrical studies of Polyaniline magnesium cobalt ferrite composites, Megha R et al. [20] explored the electrical conductivity on polypyrrole magnesium ferrite composites. Joulaei M [21] studied the magnetic properties of magnesium ferrite composites with different polymers.

Previously, very scanty work has been conducted on the investigation of Polyaniline—Magnesium ferrite composites. Bashira A [22] reported on the efficiency of Polyaniline—magnesium ferrite composite in which magnesium ferrite was prepared by coprecipitation method and incorporated into Polyaniline matrix by in-situ polymerization and studied for photodegradation. Khafagy RM [23] prepared the magnesium ferrite powder by combustion technique and studied the magnetic properties of Polyaniline—magnesium ferrite composite. However, the novelty of our work involves the synthesis of highly homogenous porous powder of Magnesium nano ferrite by single step low-temperature self-propagating solution combustion method. The composites of Polyaniline—ferrite has both magnetic and conducting properties. A detailed analysis of linear optical assets such as absorbance and indirect and direct band gap were studied for 10%, 30% and 50% weight percentage of PANI—MFe_2_O_4_ composites. The decrease in optical band gap with the addition of ferrites, which can be optimized and harnessed in optical applications. The dielectric properties hint at the utility of these composites in energy storage applications, as the composites show high dielectric strength and low dielectric losses [24].

## 2. Experimental Method

Materials: All chemicals of AR grade were procured from Otto Chemicals (Mumbai)

### 2.1. Synthesis of Magnesium Ferrite Nanoparticles

Magnesium nano-ferrite was prepared by low-temperature Solution combustion method. The weight percentage of nitrate is calculated by the formula:

$$Wt\;\%\;of\;nitrate=\frac{Molarity\times Molecular\;weight\times Volume}{1000}$$

Metal nitrates, i.e., Iron nitrate nonahydrate (Fe(NO_3_)_2_·9H_2_O) and magnesium nitrate hexahydrate (Mg(NO_3_)_2_·6H_2_O), are taken in the molar ratio of 1:2 in 100 mL deionized water. The oxidizing valency of metal nitrates is balanced by reducing the valency of the fuel urea, and thus we obtain 6.66 moles of urea to be added to the solution of metal nitrates. The solution is stirred well to obtain a clear homogenous solution and placed in a silica crucible. The solution is heated in the Muffle furnace for up to 500 °C to obtain fine voluminous magnesium ferrite nano-powder, which is crushed in agate and mortar pestle and then calcined for 3 h at 300 °C.

### 2.2. Synthesis of Composites

**Figure 1:**
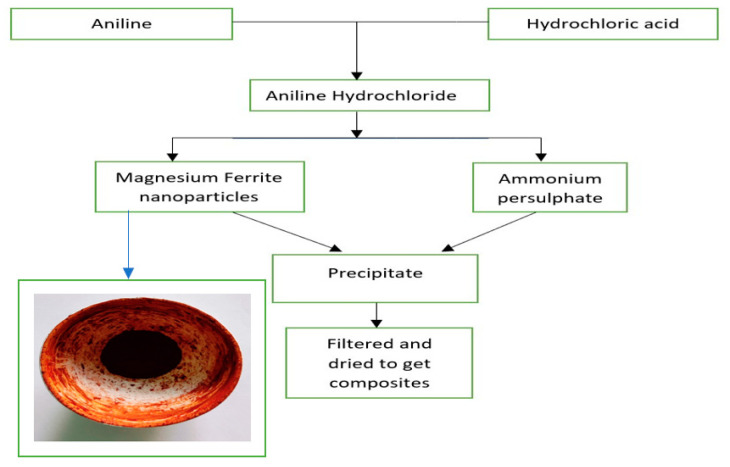
Schematic representation of the method of synthesis of Polyaniline—Magnesium ferrite composites.

100 mL of 1 N HCl is taken in a beaker, and to this, the Aniline monomer is added to obtain aniline hydrochloride. The beaker is kept in an ice bath maintained at 0–5 °C, and the oxidizer, Ammonium persulphate, is added drop by drop continuously to the aniline hydrochloride solution till the solution colour changes from brownish to dark green. The solution is stirred incessantly and placed for the process of polymerization for 4 h. The solution was further kept in the freezer for 24 h. Whatman filter paper was used to filter the solution, and the resultant precipitate obtained was washed with distilled water and acetone multiple times to get rid of impurities. The obtained precipitate was first air-dried and then dried in a hot air oven at 60 °C. Polyaniline as the final product was obtained. The prepared Nano-ferrite powder is added in different weight percentages (10%, 30% and 50% w.r.t aniline monomer) during the polymerization of Polyaniline by above-mentioned method to obtain different wt. % of Polyaniline Magnesium ferrite composites as reported in our earlier work [25], the schematic presentation of the same is shown in Figure 1.

### 2.3. Materials Characterization

The crystal structure identification of the powdered samples was carried out with an X-ray diffractometer (Rigaku miniflex) with a monochromatic CuK_α_ radiation source in the 2θ range of 10–80° with a scan rate of 4°/min. To study the dielectric properties of the samples, pellets are prepared with a hydraulic press and silver paste is used to make contacts on either side for electrical measurements. The dielectric data are obtained with PC based LCR meter (HIOKI 3532-50 HITESTER) as a function of frequency spanning from 50 Hz to 5 MHz at room temperature. The optical absorption data were recorded with a double-beam monochromatic T90+ UV-Visible spectrophotometer in the wavelength range of 200–800 nm at room temperature.

## 3. Results and Discussion

### 3.1. XRD Analysis

**Figure 2:**
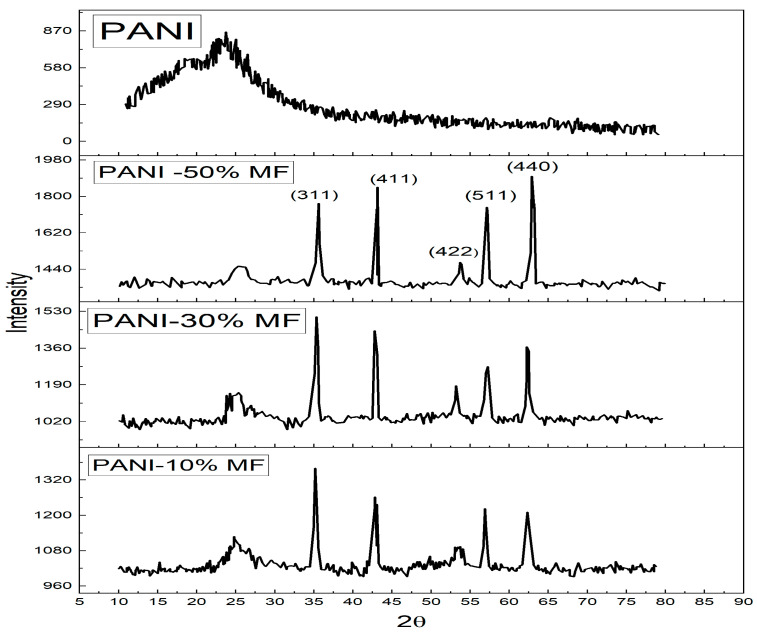
XRD Spectrum of PANI and PANI—Magnesium Ferrite (MF) Composites.

The XRD pattern of the composites is given in Figure 2. Polyaniline has an amorphous nature with a broad peak at a 2θ angle of 25° [26]. The sharp peaks are due to Magnesium nano ferrite particles at 2θ angles of 35.6°, 43°, 53.7°, 57.4° and 62.8° and could be indexed to (hkl) planes of (311), (411), (422), (511) and (440) thus confirming the formation of composite [27]. The spectra did not show any other peaks for impurities. The Magnesium Ferrite peaks become more intense in the composite as Magnesium ferrite content increases in composites and the broad peak of Polyaniline diminishes in intensity. The same result was observed by Özlem Yavuz et al., that the addition of ferrite increased the crystallinity of the composites [28]. The average crystallite size of all samples is estimated for the most intense (311) peak using Debye–Scherrer formula [29] given as Equation (1)

(1) $$D=\frac{k\lambda }{\beta \cos\theta },$$

where *λ* is the wavelength (1.54 A°), *D* is the crystallite size, *k* is the Scherrer constant with a value of 0.94, and *β* is the FWHM (Full-width half maxima) of the most intense peak. The calculated crystallite sizes are 14 nm, 16 nm and 23 nm for 10%, 30% and 50%, respectively [30].

**Figure 3:**
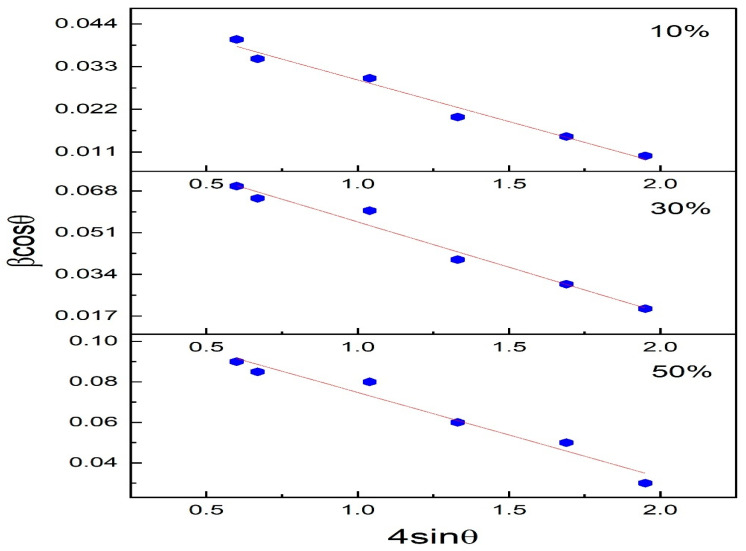
Williamson hall plot of Polyaniline-magnesium ferrite composites (10%, 30% and 50%).

Williamson–Hall (WH) plot enables us to consider the line broadening of XRD spectra due to the crystallite size as also due to the strain in the crystal structure [31]. The modified Williamson—Hall method considers that there will be a uniform distribution of strain in all the crystallographic directions [32]. The broadening of a peak can be expressed as Equation (2) [33]

(2) $$\beta \cos\theta =\frac{k\lambda }{D}+4\varepsilon \sin\theta$$

here in Equation (2), *β* is the FWHM (Full-width half maxima) of the peaks, and *ε* is the intrinsic strain in the crystal. Equation (2) is the equation of a straight line. If 4sin *θ* is plotted against βcos θ, the intrinsic strain (ε) is given by slope and from the intercept, the size of the crystallite can be evaluated. Size of the crystallite of Polyaniline—Magnesium Ferrite (PANI—MF) (10%), PANI—MF) (30%), PANI—MF) (50%) were estimated to be 12.64 nm, 13.15 nm and 20.12 nm, respectively, by calculating the slope of *β*cos*θ* vs. 4sin*θ* as shown in Figure 3. These values agree with that obtained by the Scherrer formula. The composites of PANI—MF (10%), PANI—MF (30%) and PANI—MF (50%) have intrinsic strain values of 3.61 × 10^−4^, 5.05 × 10^−4^ and 8.75 × 10^−4^ respectively. The values of lattice strain show that there is lattice expansion. The calculated values are given in Table 1.

### 3.2. Optical Properties

**Figure 4:**
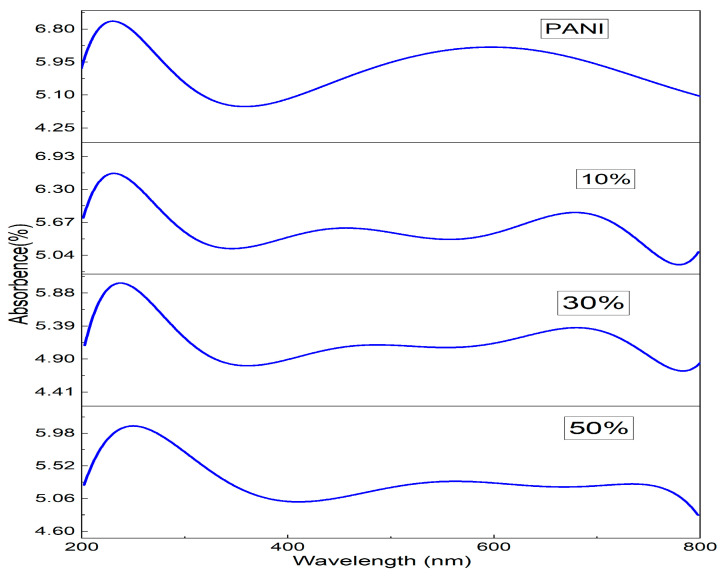
UV-V is the absorption spectra of PANI and PANI-MF composites.

**Figure 5:**
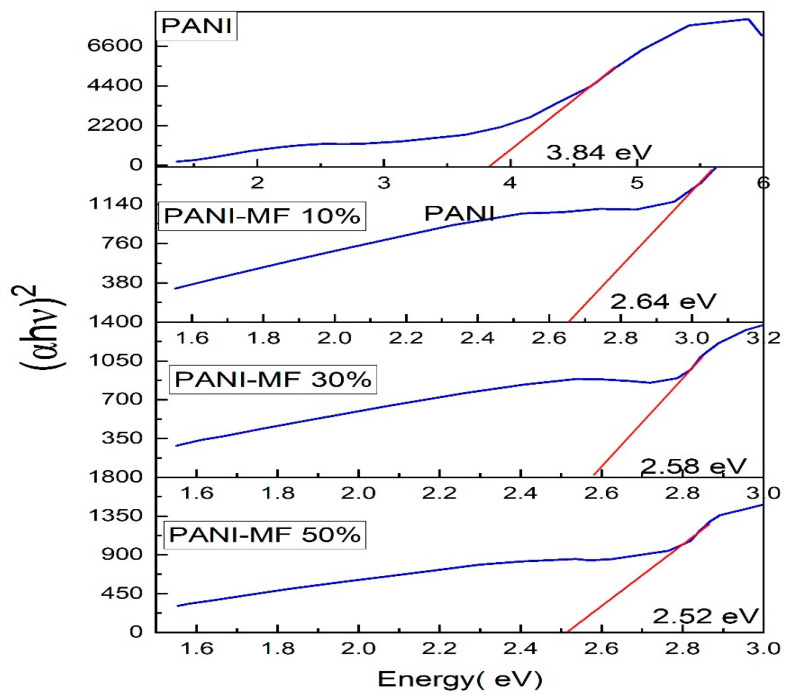
Direct band gap of composites.

**Figure 6:**
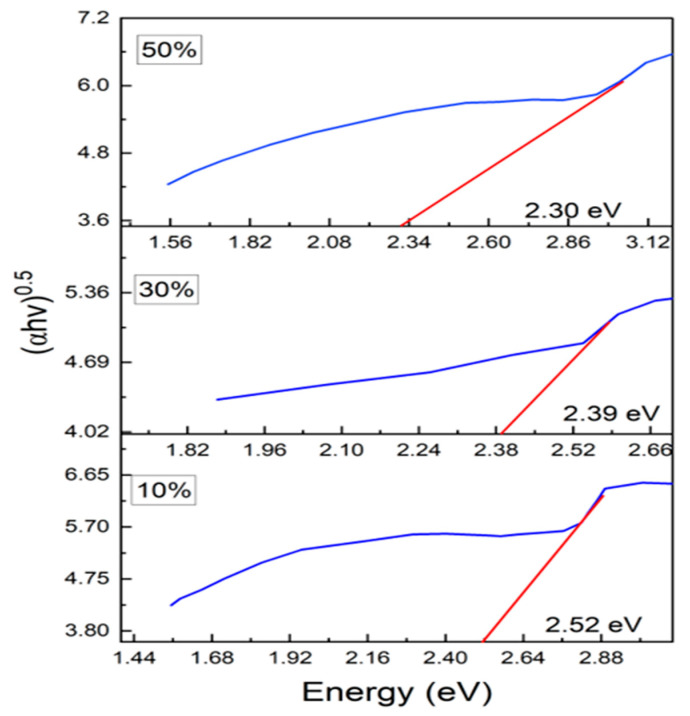
Indirect band gap of the composites.

**Figure 7:**
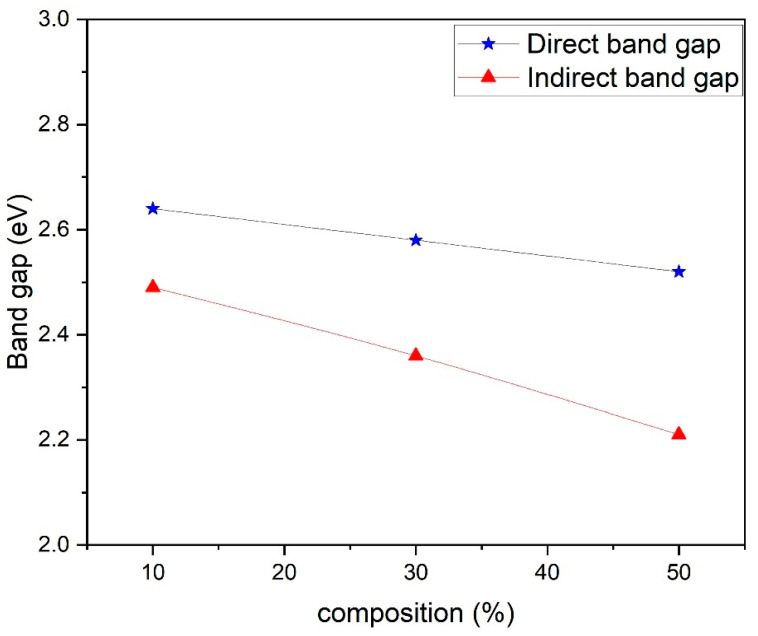
Variation of direct and indirect band gap in different composites.

The UV-Visible absorption spectra of PANI and PANI—Magnesium Ferrite (MF) composites are shown in Figure 4. The absorption spectrum of PANI shows two absorption peaks at wavelengths of 230 nm and 591 nm. The 230 nm peak is due to π–π* transition in the phenyl ring, and the 591 nm absorption peak is due to the charge transfer from the HOMO of the benzenoid ring to the LUMO of the quinoid ring [34]. In the composites, the first peak occurs at 230, 238 and 255 nm, respectively, for 10%, 30% and 50% composites showing an increase in the wavelength. The peak at 591 nm in Polyaniline appears to be shifted to the higher wavelength at 682 nm, 690 nm and 750 nm in composites of 10%, 30% and 50%. Both redshifts in the peaks indicate that lower energy is required for the transitions in the composites than that of Polyaniline. We see extra peaks in the composites at wavelengths of 456 nm, 465 nm and 539 nm, which is due to the magnesium ferrite nanoparticles in the composite. When UV-Vis light is incident on a material, there will be a transition of electrons from the valence band to the conduction band, and if, during the transition, the momentum of the electron remains the same in both ground and excited states, it is a direct transition, and if the momentum changes during the transition along with the emission of phonon then it is an indirect transition. The optical band gap is calculated with the Tauc equation given as Equation (3)

(3) $$\left(\alpha h\nu \right)=A{\left(hv-E_{g}\right)}^{n},$$

where *n* is an index, *Eg* is the optical band gap, *A* is a constant dependent on the transition probability, and *h* is Planck’s constant, which is theoretically taken to be two for direct allowed transition and a value of 1/2 for indirect allowed transition. For the determination of the direct band gap, the linear region is extrapolated on the x-axis on a graph of (*ahv*)^2^ plotted as a function of photon energy *hv* exposed in Figure 5. The direct band gap takes values of 3.84 eV for PANI, and in the composites, the values of the optical band gap decrease as the percentage of ferrite increases in the composites. The direct optical band gap takes values of 2.64 eV, 2.58 eV and 2.52 eV for 10%, 30% and 50% composites. To calculate the indirect band gap (*ahv*)^0.5^ is plotted as a function of energy and extrapolated on the x-axis, as shown in Figure 6. The indirect band gap takes values of 2.52 eV, 2.39 eV and 2.30 eV for 10%, 30% and 50% of Polyaniline—Magnesium ferrite composites. The decrease in both direct and indirect band gap as a function of composition is shown in Figure 7. The same results of a decrease in the optical band gap of Polyaniline with the addition of ferrite were observed by Swati Sehrawat et al. [35]. Tchouank Tekou et al. had the same results of a decrease in optical band gap with the addition of hexaferrite to Polyaniline [36].

### 3.3. Dielectric Properties

**Figure 8:**
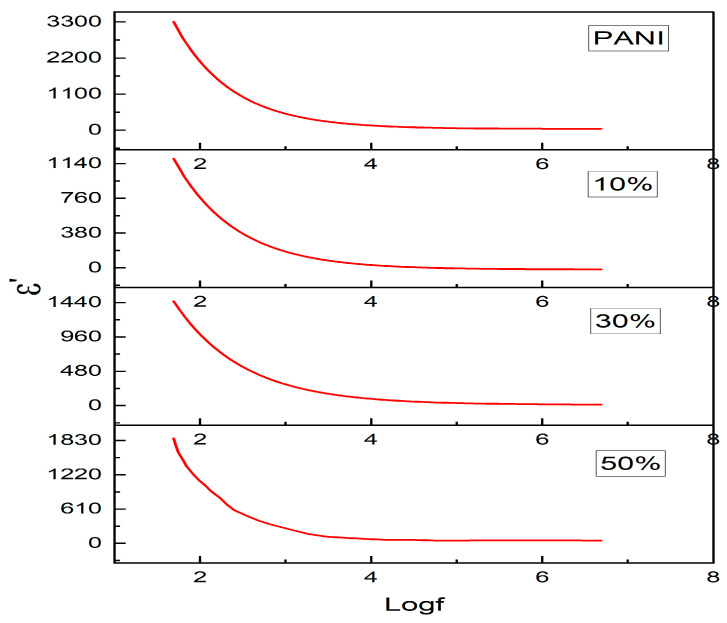
Variation of $\varepsilon^{\prime }$ in different composites.

Dielectric measurements are dependent on fillers, method of synthesis and nature of components. The complex dielectric constant can be defined as

(4) $$\varepsilon^{*}=\varepsilon^{\prime }-J\varepsilon^{\prime \prime },$$

where, $\varepsilon^{\prime }$ is the real part of the dielectric constant and measures the stored energy in a material due to polarization, and $\varepsilon^{\prime \prime }$ is the imaginary part of the dielectric constant measuring the dissipated energy in the material. $\varepsilon^{\prime }$ values are calculated from the capacitance measured at room temperature for the samples using Equation (4) spanning frequency from 50 Hz to 5 MHz

(5) $$\epsilon \prime =\frac{cd}{\varepsilon_{0}A},$$

where *C* is the measured capacitance, *A* is the area of the sample pellets, *d* is the thickness and $\varepsilon_{0}$ is the permittivity of free space (*ε*_0_ = 8.854 × 10^−12^ F/m). Figure 8 shows the variation of $\varepsilon^{\prime }$ as a function of the logarithm of frequency for Polyaniline and Polyaniline—Magnesium ferrite composites of different weight ratios. For all the samples, we see a steep decrease in $\varepsilon^{\prime }$ with frequency in the low-frequency region of less than 10^3^ Hz, and at higher frequencies, we see almost frequency-independent behavior, i.e., above a frequency of 10^3^ Hz. A dielectric material in the low-frequency region has polarization such as dipolar, atomic, interfacial and electronic that sum up to the net polarization resulting in the high value of *ε*’ [37], and for the higher frequencies, there will only be a contribution from electronic and atomic polarizations with other polarizations becoming ineffective and thus decrease in $\varepsilon^{\prime }$. Polyaniline shows strong polarization due to polaron/bipolaron and other bound charges, which leads to a high value of $\varepsilon^{\prime }$ at low frequency. In the composites, the high values of the $\varepsilon^{\prime }$ at the low frequencies are due to various factors such as grain boundary defects, oxygen vacancies and Fe^+2^ ions [38]. For all the samples, we see a plateau region at high frequency which is due to the electrical relaxation process [39] wherein the dipoles fail to follow the AC field, and there will be lagging of polarization w.r.t field. With the higher doping percentage of Magnesium ferrite in the composite, the $\varepsilon^{\prime }$ is found to have higher values which can be explained according to the model given by Maxwell and Wagner. Maxwell—Wagner model considers the double-layer dielectric structure model of an inhomogeneous medium. In this model, the structure of dielectric is supposed to contain grain boundaries and grains. In low-frequency regions, the grain boundaries are dominant, providing high resistance and high. $\varepsilon^{\prime }\;$values, and in high-frequency regions, the effect of grains is dominating, giving almost linear permittivities [40].

**Figure 9:**
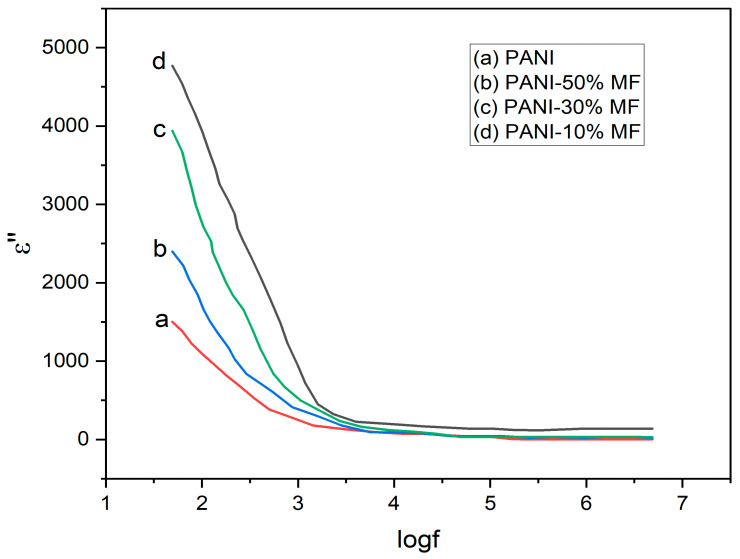
Variation of $\varepsilon^{\prime \prime }$ as a function of log⁡ f.

The dielectric loss $(\varepsilon^{\prime \prime })\;$as a function of frequency is plotted in Figure 9, and it has similar nature as that of the dielectric constant. Dielectric loss measures the amount of energy dissipated in the dielectric as a function of the applied electric field. The dielectric loss also declines with increasing frequency, and it becomes almost independent of frequency after 10^3^ Hz. Again, at low frequencies, due to dominating grain boundaries, large resistivity is caused, which leads to higher dielectric loss, whereas at low frequencies, the conducting grains offering low resistance led to low values of $\varepsilon^{\prime \prime }$.

## 4. Conclusions

In the current study, a simple spinel ferrite, i.e., Magnesium ferrite nanoparticles, was prepared by low-temperature self-propagating solution combustion technique, which is an effective one-step synthesis method of ferrites. The synthesized nanoparticles are doped with Polyaniline during polymerization to obtain the composites in three weight percentages of 10%, 30% and 50% of magnesium ferrite in the polymer. XRD spectra confirm the formation of the composite. Dislocation density and micro-strain both decreased in all the samples as the percentage of Magnesium ferrite increased in the polymer matrix. UV-Vis absorption peaks of PANI are red-shifted in the composites. The direct optical band gap is determined with a Tauc plot, and the direct band gap values decrease as the percentage of Magnesium ferrite increases in the samples from 3.84 eV for PANI to a value of 2.52 eV for PANI—Magnesium Ferrite 50% composite. The indirect band gap is seen least for 50 % composite with a value of 2.30 eV. A study of dielectric data reveals that the dielectric constant, as well as dielectric loss, decreases as a function of increasing frequency up to 10^3^ Hz, and for higher frequency regions, they appear to be nearly independent of frequency. Thus, we see that the material of interest analyzed displays the systematic variation in size, energy gap and dielectric properties, which provides for its applications in sensors, as a corrosive coating, energy storage application etc.

## Figures and Tables

**Table 1:** Crystallite size and Lattice strain of Polyaniline—Magnesium ferrite composites.

Sample	Crystallite Size(D) nm (Calculated from Debye Scherrer Formula)	Crystallite Size(D) nm (Calculated from W-H Plot)	Lattice Strain (ϵ) ×10^−3^
PANI-10% Magnesium Ferrite	14	12.64	2.57
PANI-30% Magnesium Ferrite	16	13.15	2.25
PANI-50% Magnesium Ferrite	23	20.12	1.56

## Data Availability

Data availability on request.
